# Identity in interaction: momentary dynamics of self-appraisal and reflected appraisal

**DOI:** 10.3389/fpsyg.2026.1814576

**Published:** 2026-07-15

**Authors:** Isabel Dorothee Gütges, Haoran Xi, Siegfried Gauggel, Saskia Doreen Forster

**Affiliations:** Institute of Medical Psychology and Medical Sociology, University Hospital RWTH Aachen, Aachen, Germany

**Keywords:** ecological momentary assessment, meta-perception, mood, reflected appraisal, self-appraisal, appraisal discrepancy, social context

## Abstract

**Introduction:**

A coherent sense of self depends not only on how individuals evaluate themselves, but also on how they believe they are evaluated by others. These internal and socially inferred self-evaluations—self-appraisal and reflected appraisal—are central to identity regulation and emotional wellbeing, yet their momentary interplay in everyday life remains poorly understood. In particular, little is known about how self- and reflected appraisal are associated with one another across moments, how social context shapes their coupling, and whether discrepancies between them carry affective consequences.

**Methods:**

Using ecological momentary assessment, student participants reported their self-appraisal, reflected appraisal, social context, and mood eight times daily over a 10-day period.

**Results:**

Self-appraisal and reflected appraisal were strongly associated within the same moment, with concurrent associations substantially larger than lagged effects. Social context selectively moderated the momentary association from self-appraisal to reflected appraisal, such that self-appraisal was more strongly associated with reflected appraisal when individuals were with others compared to when they were alone, whereas the reverse association was not moderated by social context. Greater discrepancy between self-appraisal and reflected appraisal was initially associated with lower concurrent mood but was not associated with subsequent mood. Moreover, the concurrent discrepancy association was no longer significant after accounting for self-appraisal and reflected appraisal directly. Additional analyses suggested that mood was associated more strongly with self-appraisals than with discrepancies between self-appraisal and reflected appraisal.

**Discussion:**

Together, the findings indicate that self- and reflected appraisal are closely coupled yet distinguishable processes that show substantial momentary covariation in daily life. Social context was associated with stronger coupling between self-appraisal and reflected appraisal, although additional decomposition analyses suggested that this effect was not driven by within-person fluctuations in social engagement. Strong associations between self-appraisal and reflected appraisal were evident even when individuals were alone, consistent with the notion that representations of others’ evaluations remain psychologically relevant outside of direct social interaction. At the same time, the findings provide limited support for discrepancy as an independent correlate of mood, suggesting that momentary affect may be associated more strongly with the positivity or negativity of self-appraisals and reflected appraisals than with discrepancies between them. These results contribute to a more nuanced understanding of how internal and socially inferred self-evaluations are integrated in everyday life.

## 1 Introduction

In everyday social life, individuals continuously navigate two evaluative perspectives: how they see themselves and how they believe they are seen by others. These evaluations are rarely made explicit, yet they shape momentary feelings, expectations, and behavior in subtle but consequential ways. Understanding how individuals integrate their own self-evaluations with inferred social evaluations is therefore central to theories of the social self and self-concept. Two evaluative processes are particularly fundamental in this regard: self-appraisal, which captures evaluations of one’s own attributes, and reflected appraisal, which reflects how individuals think they are viewed by others. Classic symbolic interactionist accounts conceptualize these appraisals as identity-relevant meanings that emerge through social interaction and shape how individuals understand and regulate themselves in everyday life (Cooley, 1902; Natanson, 2012). Contemporary approaches similarly view self-appraisal and reflected appraisal as interdependent components of short-term evaluative processing embedded within social cognition (Srivastava and Beer, 2005; Swann, 2012). Across these perspectives, a shared assumption is that integrating internal self-evaluations with socially inferred evaluations supports a coherent and predictable sense of self.

The motivation to maintain such coherence in the moment has been emphasized across theories of self and identity (Campbell et al., 1996; Swann, 2012). A coherent self-concept facilitates predictability in experience and behavior, enabling individuals to anticipate social outcomes and regulate themselves accordingly. Self-verification theory suggests that individuals rely on their immediate self-views when interpreting ambiguous or uncertain social cues, such that momentary self-appraisal shapes reflected appraisal (Swann, 2012; Swann and Bosson, 2010). At the same time, classic reflected appraisal and symbolic interactionist models propose that individuals incorporate their inferences about how others regard them into their own self-evaluations, implying influence in the opposite direction as well (Cooley, 1902; Kinch, 1963; Srivastava and Beer, 2005). From this perspective, self-appraisal and reflected appraisal are not independent evaluative processes but are expected to be closely linked within everyday experience, reflecting the joint role of internal and socially inferred evaluative information in identity regulation.

However, mutual influence does not imply equivalence. Laboratory research on meta-perceptions, i.e., beliefs about how one is perceived by others, demonstrates that individuals are sensitive to the fact that others’ evaluations do not simply mirror their own self-views. For example, Carlson et al. (2011) showed that meta-perceptions of personality traits are more strongly correlated with others’ actual appraisals than are self-appraisals, indicating that individuals recognize that they are seen differently by others. Thus, self-appraisal and reflected appraisal are expected to covary within moments while remaining conceptually and empirically distinguishable processes. Their alignment reflects integration rather than collapse into a single evaluative representation.

Further research on meta-perception provides a cognitive account of how this momentary coupling may arise in everyday life. Meta-perceptions are constructed through mentalizing processes that integrate ambiguous social cues, contextual information, and one’s own self-view (Laing et al., 1966; Stendel and Chavez, 2025). Because others’ evaluations are not directly observable and real-time social information is often ambiguous, individuals frequently rely on their current self-appraisals as an inferential anchor when constructing beliefs about others’ evaluations, updating these beliefs only when sufficiently informative social input becomes available (Luyten and Fonagy, 2015).

Empirical evidence supports this inferential anchoring process. Meta-perceptions are systematically biased toward individuals’ own self-views and reflect self-projection more strongly than others’ actual evaluations (Srivastava and Beer, 2005; Carlson et al., 2011). For example, Srivastava and Beer (2005) showed that when participants judged how they believed others saw them, these judgments largely reflected their own self-views rather than their interaction partners’ actual evaluations. At the same time, meta-perceptions do not only reflect self-views, they also shape them. For instance, meta-perceptions of delinquency predict individuals’ delinquent self-views and subsequent behavioral outcomes (Walters, 2016). Experimental studies further demonstrate that meta-perceptions mediate the influence of others’ evaluations on self-views (Carlson et al., 2011; Yue et al., 2021). Together, these findings indicate that self-appraisal and reflected appraisal may be linked through reciprocal inferential processes and support their close coupling in daily life.

However, the strength of the momentary coupling between self-appraisal and reflected appraisal is unlikely to be invariant across situations. Social contexts are characterized by heightened evaluative salience, increased ambiguity of social cues, and the need to interpret others’ reactions in real time (Wallace and Tice, 2012; Griffin and Ross, 1991). Under such conditions, identity-relevant information may become more consequential, and both internal self-views and inferred social evaluations are likely to exert stronger influence.

Social context fundamentally alters the evaluative environment in which self-appraisal and reflected appraisal are constructed. When others are present, evaluative cues become more salient, yet these cues are rarely explicit and often ambiguous, requiring interpretation rather than direct inference (Wallace and Tice, 2012; Stendel and Chavez, 2025). Under such conditions, multiple identity-relevant processes are likely to be engaged. From a self-verification perspective, ambiguous social feedback increases the likelihood that individuals interpret others’ reactions in ways that are consistent with their existing self-views, thereby strengthening the momentary influence of self-appraisal on reflected appraisal (Swann, 2012; Swann and Bosson, 2010). Complementing this motivational account, meta-perception research suggests that individuals rely on their own self-views as an inferential anchor when constructing beliefs about how they are perceived, particularly under uncertainty (Srivastava and Beer, 2005; Carlson et al., 2011).

At the same time, symbolic interactionist and reflected appraisal accounts emphasize that social interaction provides particularly salient input about how one is viewed by others, positioning reflected appraisals as a key mechanism through which self-evaluations are shaped (Cooley, 1902; Kinch, 1963). Consistent with Sociometer Theory, which conceptualizes self-evaluations as an internal monitor of perceived social acceptance (Leary, 2012), inferred social evaluations carry heightened relevance for social acceptance and interaction outcomes when others are present. Accordingly, reflected appraisals may exert stronger momentary influence on self-appraisal in social contexts than when individuals are alone. Taken together, these perspectives converge on the expectation that social context amplifies the momentary coupling between self-appraisal and reflected appraisal.

Despite their close coupling, self-appraisal and reflected appraisal draw on distinct informational sources. Self-appraisals reflect internal cognitive states derived from self-knowledge and affect (Markus and Wurf, 1987), whereas reflected appraisals are constructed from social cues that are often ambiguous, inconsistent, or misinterpreted (Carlson et al., 2010; Carlson, 2013). As a result, momentary alignment does not imply perfect correspondence. Instead, self-appraisal and reflected appraisal typically move together, with informative deviations emerging when internal and socially inferred evaluative inputs diverge.

Identity-regulation theories propose that such misalignment carries functional significance. In Identity Control Theory, negative affect arises when perceived identity-relevant meanings fail to match, serving as an internal error signal that motivates regulatory adjustment (Stets and Burke, 2025). This logic aligns with cognitive dissonance theory (Festinger et al., 1954; Harmon-Jones and Mills, 2019) and self-discrepancy theory (Higgins, 1987), both of which link evaluative inconsistency to discomfort and reduced wellbeing. From this perspective, discrepancies between self-appraisal and reflected appraisal are not mere noise but meaningful signals that may carry immediate affective consequences. Because difficulties in emotion regulation, interpersonal sensitivity, and self-concept stability are increasingly understood as transdiagnostic processes, understanding the normative patterns of self–social evaluative integration may inform how such vulnerabilities emerge and are maintained.

Investigating self-appraisal and reflected appraisal simultaneously in daily life offers insight into the normative patterns of identity regulation, revealing how individuals integrate internal and social evaluative information, adjust to situational demands, and respond to evaluative conflict in real time. Ecological momentary assessment (EMA) provides a uniquely suitable approach for capturing these processes, as both self-appraisal and reflected appraisal show substantial within-person variability in everyday life (Gütges et al., 2025). Building on theoretical accounts of self-verification, self-concept coherence, and social evaluation, the present study adopted a theory-guided approach to examine momentary associations between self-appraisal, reflected appraisal, social context, discrepancy, and mood in daily life. Specifically, the analyses examined whether self-appraisal and reflected appraisal would show concurrent alignment in daily life, whether their momentary association would vary as a function of social context, and whether greater discrepancy between self-appraisal and reflected appraisal would be associated with lower momentary mood.

## 2 Methods

The study was conducted as part of a larger research project on momentary self-evaluative processes in daily life. Detailed information regarding recruitment process and demographic composition of the sample is provided in Gütges et al. (2025). The study was preregistered at OSF prior to data analysis (osf.io/dn6bh).

### 2.1 Participants

Participants were recruited between May and June 2023 via on-campus flyers and email invitations sent to individuals who had previously participated in departmental studies and consented to receive further invitations. To ensure adequate statistical power, *a priori* power calculations were conducted using the PowerCurves tool (Kleiman, 2021). Following recommendations for intensive longitudinal designs (Bolger et al., 2011; Maas and Hox, 2005), the calculations incorporated the planned 10-day assessment period with up to eight measurements per day. Based on these assumptions, a target sample size of approximately 100 participants was considered sufficient to detect medium-sized within-person effects with approximately 80% power.

Eligible participants were German-speaking adults between 18 and 50 years of age who owned an Android smartphone compatible with the MovisensXS application (Movisens GmbH, Karlsruhe, Germany) used for data collection. Individuals who self-reported a current neurological or psychiatric condition were excluded.

A total of 100 individuals provided written informed consent to participate. One participant discontinued early and was excluded from analysis. The final sample comprised 99 participants (53.5% male, 45.5% female, 1% diverse) aged 18–44 years (*M* = 25.1, *SD* = 4.7). Among them, 84.9% were students, 24.3% of whom were also employed, while 15.2% were employed without concurrent studies.

### 2.2 Procedure

Interested individuals were screened via email and subsequently invited to an in-person briefing session. During this session, they received comprehensive information about the study protocol and provided written informed consent. The study was approved by the local ethics committee (Protocol EK 22-325) in accordance with the Declaration of Helsinki. Participants were instructed to download the EMA app MovisensXS (version 1.5.23) on their smartphones and were then trained on how to respond to the daily assessments.

Data collection commenced the day following the training and continued for 10 consecutive days. Participants received eight randomized prompts per day between 8:00 a.m. and 10:00 p.m., with a minimum interval of 30 min between prompts. Each prompt was accompanied by a sound and vibration notification. If immediate response was not possible, participants could delay the response by 5, 10, or 15 min. After 20 min, the prompt expired. Each prompt could be attempted up to five times.

Responses were entered directly into the MovisensXS app and securely transmitted via encrypted connection to a central database. Data were monitored throughout the study for completeness and technical issues, with in-app chat support available for troubleshooting. Participants did not receive reminders for missed assessments but were periodically informed about their completion rate. Missed prompts were not repeated.

Compensation was performance-based: participants received €35 for at least 50% completion, €50 for ≥75%, and €75 for ≥90% completion.

### 2.3 Measures

All measures were administered in German via MovisensXS (App version 1.5.23; Movisens GmbH, Karlsruhe, Germany). The original German items, English translations, and additional variables from the broader project are available in the Supplementary Material of Gütges et al. (2025).

#### 2.3.1 Ecological momentary assessments

The EMA protocol consisted of 15 items divided into four subsets presented in fixed order, except for the self-appraisal and reflected appraisal items, whose order alternated randomly at each prompt to minimize sequence effects.

##### 2.3.1.1 Mood

Following the framework of Wilhelm and Schoebi (2007), momentary mood was conceptualized along three intercorrelated dimensions: valence, energetic arousal, and tensed arousal. Valence was assessed with an EMA adaptation of Simons et al. (2020), including three positive affect items (content, happy, cheerful) and four negative affect items (insecure, down, guilty, afraid). Items were rated on a 7-point Likert scale (0 = not at all, 6 = very). Energetic arousal and tensed arousal were each measured using one bipolar item rated on the same scale (0 = without energy to 6 = full of energy; 0 = very tense to 6 = very relaxed). For each prompt, mean scores were computed for the three facets, which were then averaged into a composite mood index representing overall affective state (Wilhelm and Schoebi, 2007).

##### 2.3.1.2 Self-appraisal and reflected appraisal

Self-appraisal and reflected appraisal were each assessed using four items derived from the German version of the Rosenberg Self-Esteem Scale (Von Collani and Herzberg, 2003), as adapted in prior EMA studies (Santangelo et al., 2017, 2020). Self-appraisal was measured with items such as “I am satisfied with myself” and “I consider myself a person of worth.” Reflected appraisal items mirrored this structure, e.g., “Others are satisfied with me” and “Others consider me a person of worth.” Items were rated on a 10-point Likert-scale ranging from 0 (does not apply at all) to 9 (fully applies). Internal consistency was good (SA: *α* = 0.81; RA: *α* = 0.80). Within-person reliability was good for both self-appraisal (*α* = 0.81) and reflected appraisal (*α* = 0.80). Between-person reliability was excellent for self-appraisal (*α* = 0.92) and reflected appraisal (*α* = 0.94). Also, strong convergent validity was observed for the self-appraisal item (Spearman *r* = 0.77 with the Rosenberg Self-Esteem Scale), suggesting alignment with trait self-esteem while reflecting state-specific variation.

##### 2.3.1.3 Social context

Social context was assessed using the item “at this moment I am …” and measured with the response options “alone” and “in contact with others”. Participants could only choose one of the two response options.

### 2.4 Data analysis

All analyses were performed in R (version 4.3.2; R Core Team, 2020; RRID:SCR_001905) using RStudio (RStudio Team, 2024; RRID:SCR_000432) and the packages psych (Revelle, 2018), lme4 (Bates et al., 2014), and Hmisc (Harrell and Dupont, 2020).

#### 2.4.1 Data preparation

Composite scores for self-appraisal, reflected appraisal, valence, tense arousal, and energetic arousal were computed as the mean of each item set. Negatively worded items (e.g., negative affect, reverse-coded self-appraisal and reflected appraisal) were recoded so that higher values indicated more positive states (e.g., more positive self-evaluation, better mood). An absolute discrepancy variable |SA {*t*} - RA {*t*}| was computed.

#### 2.4.2 Analyses

All analyses were preregistered as exploratory and are interpreted as theory-informing rather than theory-testing. To examine the momentary relationships between self-appraisal, reflected appraisal, and mood, a series of multilevel regression analyses focusing on within-person processes was conducted. Separate analyses were performed in which self-appraisal and reflected appraisal were alternately entered as predictors and outcomes at the same measurement occasion. Additional analyses examined whether social context moderated the concurrent associations between self-appraisal and reflected appraisal and whether discrepancies between self-appraisal and reflected appraisal were associated with mood concurrently and across adjacent assessments.

All time-varying predictors were person-mean centered, but not standardized, to capture within-person fluctuations relative to each participant’s typical level. For social context, within-person and between-person components were separated. Participants’ average proportion of social situations across the study period captured between-person differences, whereas momentary deviations from this average represented within-person fluctuations in social context.

Lagged predictors (RA {*t* − 1}, SA {*t* − 1}, discrepancy {*t* − 1}, and mood {*t* − 1}) were created by shifting each participant’s time series forward by one assessment. Only assessments that were directly consecutive within the same day were included in lagged analyses to ensure a consistent temporal structure. Because prompt intervals varied across the day, lagged effects reflect variable temporal distances.

Primary analyses were initially specified with random intercepts and random slopes for focal within-person predictors. Several models produced singular-fit warnings, boundary estimates of random-effects parameters, or convergence problems. Therefore, more parsimonious random-intercept specifications were retained for the primary analyses. The corresponding random-slope models are reported in the Supplementary Material and yielded highly similar fixed-effect estimates.

Confidence intervals were computed using Wald-based estimation, and statistical inference was based on parameter estimates, standard errors, confidence intervals, and associated t statistics. Models were estimated using all available observations, with missing observations handled through maximum likelihood estimation inherent in multilevel modeling.

##### 2.4.2.1 Momentary associations between self-appraisal and reflected appraisal

To investigate the concurrent relationship between self-appraisal and reflected appraisal, separate multilevel analyses were conducted. In the first analysis, reflected appraisal was entered as the dependent variable and self-appraisal served as the predictor, while RA {*t* − 1} was included to control for autoregressive stability. In the second analysis, self-appraisal was entered as the dependent variable and reflected appraisal served as the predictor, while SA {*t* − 1} controlled for stability. These analyses examined whether momentary fluctuations in self-appraisal and reflected appraisal were closely aligned beyond each construct’s prior level.

##### 2.4.2.2 Moderation by social context

To examine whether the momentary relationship between self-appraisal and reflected appraisal varied as a function of social context, moderation analyses were conducted using multilevel regression models. Social context (alone vs. with others) was entered as a person-mean centered level-1 moderator of the concurrent associations between self-appraisal and reflected appraisal.

Two separate models were estimated. In the first model, reflected appraisal served as the dependent variable, with momentary self-appraisal, social context, and their interaction entered as predictors. RA{*t* − 1} was included to control for autoregressive stability. In the second model, self-appraisal was entered as the dependent variable, with reflected appraisal, social context, and their interaction as predictors, while controlling for prior self-appraisal (SA{*t* − 1}). These models examined whether the strength of the concurrent association between self-appraisal and reflected appraisal differed depending on whether participants were alone or in a social context.

All time-varying predictors were person-mean centered, and models were estimated as multilevel regressions with random intercepts for participants to account for between-person differences. Significant interaction terms were followed up by estimating simple slopes separately for moments spent alone and moments spent with others.

Additional analyses were conducted to distinguish immediate within-person fluctuations in social context from broader between-person differences in overall social exposure. To separate within-person and between-person sources of variance in accordance with recommendations for multilevel longitudinal data (Curran and Bauer, 2011; Bolger and Laurenceau, 2013), social context was decomposed into two components: a within-person component reflecting momentary deviations from each participant’s typical level of social engagement and a between-person component reflecting each participant’s average proportion of social situations across the study period.

The moderation analyses were then repeated using the decomposed social-context variables. In the first model, reflected appraisal served as the dependent variable. Predictors included momentary self-appraisal, participants’ average level of social engagement across the study period, momentary deviations from this average level of social engagement, and the interaction between self-appraisal and momentary social-context deviations. Prior reflected appraisal was included to control for autoregressive stability. This model examined whether the association between self-appraisal and reflected appraisal was stronger during moments in which individuals were more socially engaged than was typical for them personally.

In the second model, self-appraisal served as the dependent variable. Predictors included momentary reflected appraisal, participants’ average level of social context across the study period, momentary deviations from this average level of social context, and the interaction between reflected appraisal and momentary social-context deviations. Prior self-appraisal was included to control for autoregressive stability. This model examined whether the association between reflected appraisal and self-appraisal was stronger during moments in which individuals were more socially engaged than was typical for them personally.

##### 2.4.2.3 Momentary discrepancy predicting mood

To examine the emotional consequences of divergence between self-appraisal and reflected appraisal, discrepancy was entered as the level-1 predictor in a regression model predicting mood, with mood {*t* − 1} included to adjust for prior mood. This analysis assessed whether larger momentary gaps between self-appraisal and reflected appraisal were associated with lower mood. In addition, a lagged analysis was performed to examine whether discrepancy at the previous time, predicted mood in the moment. Thus, discrepancy{*t* − 1} was entered as the level-1 predictor in a regression model predicting mood while controlling for autoregressive stability.

Additional discrepancy robustness analyses were conducted. These included multilevel models controlling directly for concurrent and lagged self-appraisal and reflected appraisal levels to examine whether discrepancy effects remained beyond the component appraisals themselves. In addition, exploratory multilevel polynomial regression analyses including linear, quadratic, and interaction terms (Edwards and Parry, 1993) of self-appraisal and reflected appraisal were conducted to further characterize their joint associations with mood and to examine whether the apparent discrepancy findings remained evident when nonlinear relations between self-appraisal and reflected appraisal were taken into account.

## 3 Results

### 3.1 Descriptive statistics

On average, participants responded to 71.7 of the 80 prompts (SD = 6.9), corresponding to a compliance rate of 89.6%, which remained stable across the assessment period. Mood showed substantial within-person variability (76%), as did self-appraisal (30%) and reflected appraisal (28.3%), supporting the analysis of momentary associations.

#### 3.1.1 Discrepancy

The mean absolute within-person discrepancy between self-appraisal and reflected appraisal was modest (*M* = 0.45), indicating that the two appraisals typically differed by less than half a scale point. Discrepancy showed substantial moment-to-moment variability (*SD* = 0.60). Across all observations, 70.7% of moments showed discrepancies below 0.50, 18.7% showed discrepancies between 0.50 and 1.00, and 10.6% showed discrepancies of one scale point or more. Person-level summaries reflected a similar pattern (*M* = 0.45, *SD* = 0.47), indicating that although self- and reflected appraisals were generally aligned, individuals regularly experienced momentary deviations between how they evaluated themselves and how they believed they were evaluated by others.

### 3.2 Self-appraisal and reflected appraisal

First, it was examined whether self-appraisal and reflected appraisal were associated with one another within the same moment while accounting for their respective autoregressive stability. When predicting reflected appraisal from momentary self-appraisal, higher momentary self-appraisal was strongly associated with higher reflected appraisal (*b* = 0.52, *SE* = 0.01, 95% CI [0.50, 0.54], *t* = 52.47). Prior reflected appraisal was also significantly associated with current reflected appraisal (*b* = 0.14, SE = 0.01, CI [0.12, 0.16], *t* = 12.83) although this effect was substantially smaller than the concurrent association.

Similarly, when predicting self-appraisal from momentary reflected appraisal, higher reflected appraisal was strongly associated with higher self-appraisal (*b* = 0.60, *SE* = 0.01, 95% CI [0.58, 0.63], *t* = 52.14). Prior self-appraisal also showed a significant positive association with current self-appraisal (*b* = 0.17, *SE* = 0.01, 95% CI [0.15, 0.19], *t* = 15.85). Thus, in both models, the concurrent associations between self-appraisal and reflected appraisal were markedly stronger than their respective lagged effects. The results of all primary multilevel models are summarized in Table 1.

**Table 1:** Summary of primary multilevel model results.

Model	Outcome	Predictor(s)	Lagged covariate	*N*	*b*	SE	95% CI	*t*
1	RA	SA	RA (*t* − 1)	5,746	0.52	0.01	[0.50, 0.54]	52.47
2	SA	RA	SA (*t* − 1)	5,746	0.60	0.01	[0.58, 0.63]	52.14
3	RA	SA × social context	RA (*t* − 1)	5,746	0.06	0.02	[0.02, 0.10]	3.12
3a	RA (alone)	SA	RA (*t* − 1)	3,063	0.48	0.01	[0.46, 0.51]	37.57
3b	RA (with others)	SA	RA (*t* − 1)	2,683	0.54	0.01	[0.51, 0.57]	36.67
4	SA	RA × social context	SA (*t* − 1)	5,746	−0.02	0.02	[−0.06, 0.03]	−0.67
4a	SA (alone)	RA	SA (*t* − 1)	3,063	0.61	0.02	[0.58, 0.64]	36.74
4b	SA (with others)	RA	SA (*t* − 1)	2,683	0.60	0.02	[0.57, 0.63]	36.70
5	Mood	Discrepancy	Mood (*t* − 1)	5,748	−0.18	0.02	[−0.21, −0.15]	−11.72
6	Mood	Discrepancy (*t* − 1)	Mood (*t* − 1)	5,748	−0.03	0.03	[−0.08, 0.03]	−1.01

All predictors were person-mean centered to isolate within-person fluctuations. Lagged predictors were based on directly consecutive assessments within the same day. Models were estimated using multilevel regression with random intercepts for participants. Models 3a–4b represent simple slopes estimated separately for moments spent alone versus with others. SA = self-appraisal; RA = reflected appraisal; CI = confidence interval.

### 3.3 Moderation by social context

**Figure 1:**
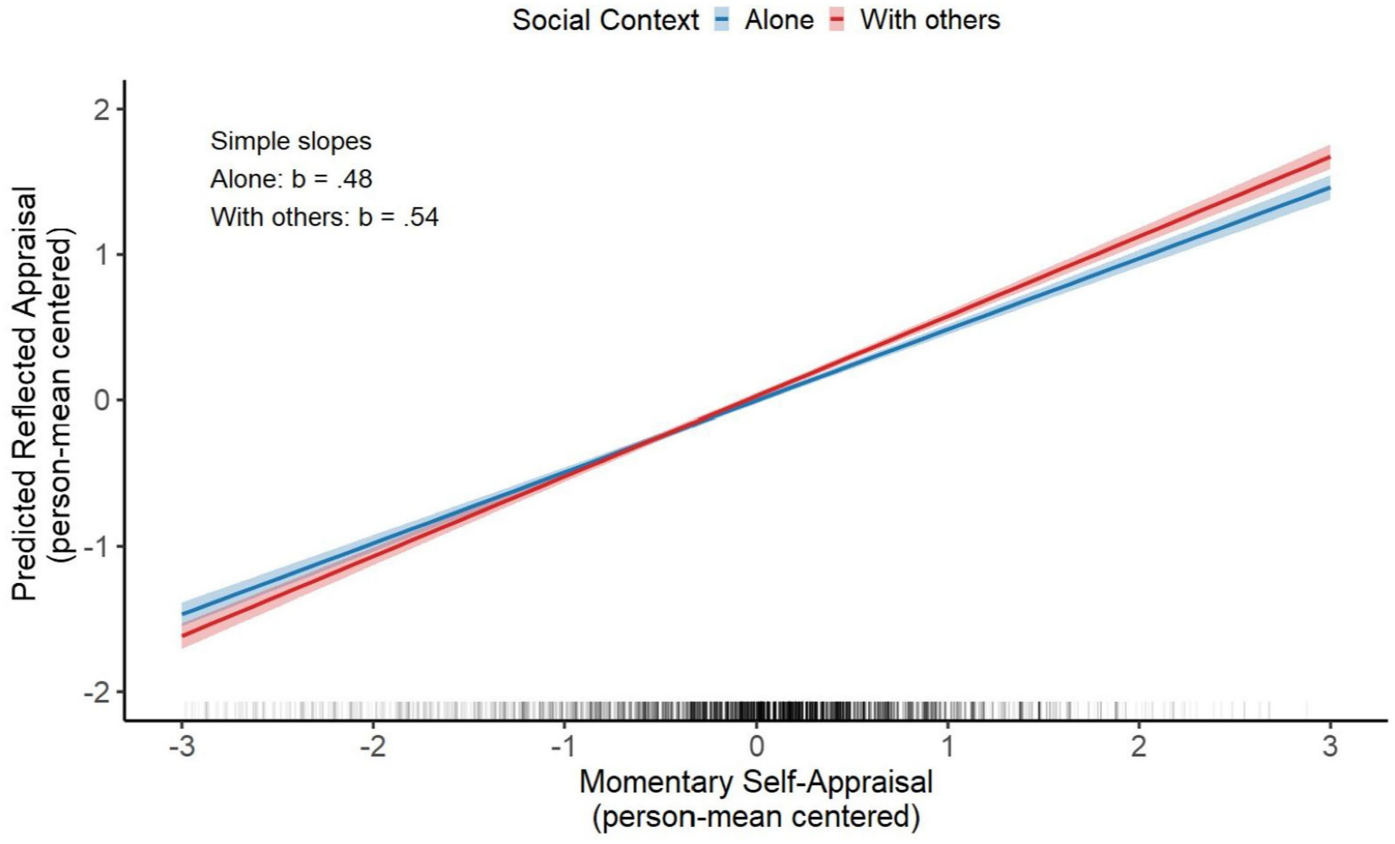
Momentary self-appraisal predicts reflected appraisal when with others or alone. Model-based predicted values of reflected appraisal as a function of momentary self-appraisal, shown separately for moments spent alone versus with others. Predictions are based on the primary multilevel moderation model including self-appraisal, social context, their interaction, and lagged reflected appraisal. Self-appraisal and reflected appraisal were person-mean centered to examine within-person associations. Lagged reflected appraisal was included as a covariate and held constant at its mean. Shaded bands represent 95% confidence intervals.

Next, it was examined whether the concurrent associations between self-appraisal and reflected appraisal differed depending on social context. When predicting reflected appraisal from momentary self-appraisal, the interaction between momentary self-appraisal and social context was significant (*b* = 0.06, *SE* = 0.019, 95% CI [0.02, 0.10], *t* = 3.12), indicating that the strength of the concurrent association varied as a function of whether participants were alone or with others (see Figure 1). When participants were alone, higher momentary self-appraisal was associated with higher reflected appraisal (*b* = 0.48, *SE* = 0.013, 95% CI [0.46, 0.51], *t* = 37.59). When participants were in a social context, this association was stronger (*b* = 0.54, *SE* = 0.014, 95% CI [0.51, 0.57], *t* = 36.69). Prior reflected appraisal remained a significant predictor (*b* = 0.14, *SE* = 0.01, 95% CI [0.10, 0.14], *t* = 12.79). Social context also showed a small but significant main effect (*b* = 0.03, *SE* = 0.02, 95% CI [0.00, 0.07], *t* = 1.87).

In contrast, when predicting self-appraisal from momentary reflected appraisal, the interaction between momentary reflected appraisal and social context was not significant (*b* = 0.01, *SE* = 0.02, 95% CI [−0.06, 0.03], *t* = 0.58,), indicating that the concurrent prediction of self-appraisal by reflected appraisal did not differ between social contexts. Higher momentary reflected appraisal predicted higher self-appraisal both when participants were alone (*b* = 0.61, *SE* = 0.02, 95% CI [0.58, 0.64], *t* = 36.75) and when participants were in a social context (*b* = 0.60, *SE* = 0.02, 95% CI [0.57, 0.64], *t* = 36.72). Prior self-appraisal remained a significant predictor (*b* = 0.17, *SE* = 0.01, 95% CI [0.14, 0.18], *t* = 15.81).

To further distinguish immediate within-person fluctuations in social context from broader between-person differences in average social exposure, additional decomposition analyses were conducted. Social context was decomposed into within-person fluctuations and between-person differences in average social exposure.

When predicting reflected appraisal, higher momentary self-appraisal was strongly associated with higher reflected appraisal (*b* = 0.51, *SE* = 0.01, 95% CI [0.50, 0.53], *t* = 51.74). Prior reflected appraisal also positively predicted current reflected appraisal (*b* = 0.14, *SE* = 0.01, 95% CI [0.12, 0.16], *t* = 12.79). The interaction between self-appraisal and within-person social context was not statistically significant (*b* = 0.03, *SE* = 0.02, 95% CI [−0.01, 0.07], *t* = 1.69), indicating that the concurrent association between self-appraisal and reflected appraisal did not reliably differ between moments spent alone and moments spent with others. Within-person social context showed a small positive association with reflected appraisal (*b* = 0.04, *SE* = 0.02, 95% CI [0.00, 0.07], *t* = 2.16), whereas between-person differences in average social exposure were unrelated to reflected appraisal (*b* = −0.02, *SE* = 0.05, 95% CI [−0.12, 0.08], *t* = −0.44).

Similarly, when predicting self-appraisal, higher momentary reflected appraisal was strongly associated with higher self-appraisal (*b* = 0.60, *SE* = 0.01, 95% CI [0.58, 0.63], *t* = 51.40), while prior self-appraisal also significantly predicted current self-appraisal (*b* = 0.17, *SE* = 0.01, 95% CI [0.15, 0.19], *t* = 15.81). The interaction between reflected appraisal and within-person social context was not significant (*b* = −0.01, *SE* = 0.02, 95% CI [−0.06, 0.03], *t* = −0.47), suggesting that the association between reflected appraisal and self-appraisal was comparable across social contexts. Within-person social context showed a small positive association with self-appraisal (*b* = 0.05, *SE* = 0.02, 95% CI [0.01, 0.09], *t* = 2.70), whereas between-person differences in average social exposure were unrelated to self-appraisal (*b* = 0.00, *SE* = 0.05, 95% CI [−0.11, 0.11], *t* = 0.02).

### 3.4 Discrepancy and mood

Finally, it was examined whether discrepancy between self-appraisal and reflected appraisal was associated with mood. Greater discrepancy was strongly associated with lower mood within the same moment (*b* = −0.18, *SE* = 0.02, 95% CI [−0.21, −0.15], *t* = −11.72). However, when self-appraisal and reflected appraisal were simultaneously included in the model, discrepancy no longer showed a significant association with mood (*b* = −0.02, *SE* = 0.01, 95% CI [−0.05, 0.01], *t* = −1.18). In contrast, both higher self-appraisal (*b* = 0.30, *SE* = 0.01, 95% CI [0.27, 0.32], *t* = 25.25) and higher reflected appraisal (*b* = 0.09, *SE* = 0.01, 95% CI [0.06, 0.11], *t* = 7.23) were independently associated with more positive mood within the same moment. Prior mood also remained significantly associated with current mood (*b* = 0.10, *SE* = 0.01, 95% CI [0.08, 0.11], *t* = 15.03).

Discrepancy did not predict mood over time: greater discrepancy at the previous measurement did not predict lower mood at the subsequent measurement after controlling for prior mood (*b* = −0.03, *SE* = 0.03, 95% CI [−0.08, 0.03], *t* = −1.01). In an additional robustness analysis controlling for prior self-appraisal and reflected appraisal, discrepancy likewise remained unrelated to subsequent mood (*b* = −0.01, *SE* = 0.03, 95% CI [−0.06, 0.04], *t* = −0.36). Beyond a strong autoregressive association of mood (*b* = 0.34, *SE* = 0.03, 95% CI [0.29, 0.39], *t* = 12.99), neither prior self-appraisal (*b* = 0.03, *SE* = 0.02, 95% CI [−0.02, 0.08], *t* = 1.32) nor prior reflected appraisal (*b* = 0.03, *SE* = 0.03, 95% CI [−0.02, 0.08], *t* = 1.30) showed significant associations with subsequent mood.

To further examine whether discrepancy was associated with mood beyond the underlying appraisal levels and configurations, exploratory multilevel polynomial regression analyses were conducted. In a model including self-appraisal, reflected appraisal, and their interaction, the interaction term was significant (*b* = 0.03, *SE* = 0.01, 95% CI [0.02, 0.04], *t* = 5.64). Higher self-appraisal (*b* = 0.31, *SE* = 0.01, 95% CI [0.29, 0.34], *t* = 27.16), higher reflected appraisal (*b* = 0.11, *SE* = 0.01, 95% CI [0.09, 0.14], *t* = 8.59), and prior mood (*b* = 0.09, *SE* = 0.01, 95% CI [0.08, 0.11], *t* = 14.81) were also positively associated with current mood.

However, in the full polynomial model additionally including quadratic terms, the interaction was no longer significant (*b* < 0.001, *SE* = 0.01, 95% CI [−0.02, 0.02], *t* = 0.05). Instead, a significant quadratic effect of self-appraisal emerged (*b* = 0.02, *SE* = 0.005, 95% CI [0.01, 0.03], *t* = 4.55), indicating that the association between self-appraisal and mood was nonlinear. The quadratic effect of reflected appraisal was not statistically significant (*b* = 0.01, *SE* = 0.007, 95% CI [−0.001, 0.03], *t* = 1.88).

## 4 Discussion

The present study examined within-day associations between self-appraisal and reflected appraisal across 10 days, the moderating role of social context in these associations, and the emotional consequences of discrepancies between self- and reflected appraisal. Together, the findings provide insight into how individuals integrate internal and socially inferred self-evaluations in everyday life. Self-appraisal and reflected appraisal were closely aligned within moments, indicating a strong correspondence between internal self-views and perceived evaluations by others. Social context selectively moderated this association, such that the link between self-appraisal and reflected appraisal was stronger when individuals were with others than when they were alone. Finally, although discrepancy between self-appraisal and reflected appraisal was initially associated with lower concurrent mood, this association was not robust after accounting for the underlying appraisal levels directly.

### 4.1 Momentary associations of self- and reflected appraisal

The close correspondence between self-appraisal and reflected appraisal has so far only been theorized and shown in laboratory studies (Carlson et al., 2011) focusing on the formation and impact of self-appraisal and reflected appraisal on self-concept. Consistent with theoretical accounts emphasizing the interdependence of internal and socially inferred self-evaluations, self-appraisal and reflected appraisal were closely aligned within moments. This pattern accords with symbolic interactionist perspectives and contemporary models of social cognition, which view self-evaluation as emerging from the integration of internal self-views and inferences about others’ evaluations (Cooley, 1902; Srivastava and Beer, 2005; Swann, 2012).

Importantly, the persistence of strong concurrent associations after controlling for autoregressive stability indicates that this alignment reflects close momentary correspondence rather than mere shared variance or temporal carryover. Self-appraisal and reflected appraisal were strongly associated, yet each showed unique concurrent associations with the other within the same moment. Thus, rather than representing redundant indicators of a single evaluative state, self-appraisal and reflected appraisal appear to function as closely coupled but conceptually distinct components of self-evaluation.

These findings refine existing models by demonstrating that identity-relevant integration unfolds at a fine-grained temporal scale in everyday life. Rather than operating only through gradual self-concept change, self–other evaluative associations appear to be continuously negotiated in real time. Future studies using finer-grained temporal resolution will be necessary to disentangle the micro-temporal sequencing of these evaluative processes.

### 4.2 Social context as a moderator of momentary SA-RA dynamics

The momentary coupling between self-appraisal and reflected appraisal varied across situations. Social context selectively moderated the direction from self-appraisal to reflected appraisal, such that momentary self-appraisal was more strongly associated with reflected appraisal when individuals were in a social context compared to when they were alone. In contrast, contrary to initial expectations, the momentary association between reflected appraisal to self-appraisal was comparable across social contexts. This asymmetric pattern indicates that social presence does not uniformly amplify self–other evaluative associations, but selectively shapes how individuals construct inferences about how they are perceived by others. Thus, rather than uniformly amplifying momentary coupling, social context produced a directional asymmetry in momentary self–other evaluative associations.

One possible interpretation of this asymmetry is that social situations increase the salience of socially evaluative self-processing more broadly. When individuals are with others, internal self-views may become more strongly linked to assumptions about how one is perceived socially, thereby strengthening the concurrent association between self-appraisal and reflected appraisal. Social interactions are often characterized by evaluative ambiguity, incomplete interpersonal feedback, and increased informational complexity (Wallace and Tice, 2012; Griffin and Ross, 1991). Under such conditions, individuals may rely more strongly on accessible internal self-views when forming assumptions about how they are perceived by others. Social situations may additionally increase the cognitive demands associated with socially evaluative processing, potentially strengthening reliance on readily accessible self-relevant information during interpersonal inference processes (Schneider et al., 2012; Srivastava and Beer, 2005; Apperly et al., 2008). Importantly, however, the present study did not directly assess cognitive load, mentalizing, or inferential processes. Future research should directly investigate the processes through which social context shapes momentary self–other evaluative dynamics.

The stronger moderation for the pathway from self-appraisal to reflected appraisal is also broadly consistent with theories suggesting that reflected appraisals are partly constructed from internal self-views rather than solely derived from external feedback (Carlson et al., 2011; Swann, 2012; Luyten and Fonagy, 2015). From this perspective, social situations may increase the tendency to draw on one’s own self-evaluations when inferring how one is perceived by others.

At the same time, additional decomposition analyses separating within- and between-person components of social context substantially attenuated the interaction effect and rendered it statistically non-significant. Specifically, the moderation was considerably weaker when examining whether individuals showed stronger self-appraisal–reflected appraisal coupling during moments in which they were more socially engaged than was typical for them personally. Thus, the decomposition analyses suggest that the observed moderation was not driven by within-person deviations from typical social engagement.

Interestingly, the primary and decomposed social-context measures may capture different aspects of social experience. Whereas the primary analyses focused on the distinction between being alone versus being with others, the decomposition analyses examined whether self–other evaluative coupling increased during moments that were more social than usual for a given individual. The findings suggest that the distinction between being alone versus being with others may capture different aspects of social experience than within-person fluctuations in social engagement.

The magnitude of the interaction effect was relatively modest. Although social context was associated with stronger coupling between self-appraisal and reflected appraisal, substantial associations between both constructs were evident across contexts. Thus, social context appeared to modulate rather than fundamentally alter momentary self–other evaluative dynamics.

Interestingly, social moments were associated with slightly higher self-appraisal and reflected appraisal. Thus, social context may be linked to more positive evaluations overall without necessarily strengthening the momentary association between both constructs. Future research should examine the mechanisms underlying these small effects.

More broadly, the findings suggest that self–other evaluative processes may not depend exclusively on immediate social presence. Strong associations between self-appraisal and reflected appraisal were evident even when individuals were alone, suggesting that representations of others’ evaluations may become internalized and remain psychologically accessible outside of direct social interactions. Rather than being activated only in social situations, reflected appraisals may constitute a relatively stable component of everyday self-evaluation, consistent with classic theories of the social self (Cooley, 1902; Natanson, 2012).

Taken together, the present findings suggest that the role of social context in self-appraisal–reflected appraisal dynamics may be more nuanced than a purely situational account would imply. Future research should examine whether similar patterns emerge across different samples and social environments and should further investigate the mechanisms through which social context shapes self–other evaluative processes in daily life.

### 4.3 Self-other discrepancy and mood

Initial discrepancy analyses indicated that greater momentary discrepancy between self-appraisal and reflected appraisal was associated with lower concurrent mood. At first glance, this finding appeared consistent with theories emphasizing the importance of evaluative coherence and consistency for psychological functioning (Swann, 2012; Higgins, 1987), suggesting that mismatches between internal self-views and socially inferred evaluations may be associated with less positive affective states in daily life.

However, additional analyses substantially qualified this interpretation. Contrary to the initial expectations, the emotional consequences of discrepancy appeared to be largely explained by the valence of the underlying appraisals themselves rather than by discrepancy per se. When self-appraisal and reflected appraisal were entered directly into the models, the discrepancy effect was strongly attenuated and no longer significant. Thus, the present findings do not provide strong support for discrepancy itself as an independent correlate of mood beyond the underlying appraisal levels. Instead, both self-appraisal and reflected appraisal remained significantly associated with mood when considered simultaneously, suggesting that the affective relevance of self–other evaluative processes may reside primarily in the evaluations themselves rather than in the degree of discrepancy between them.

Exploratory polynomial analyses further qualified this interpretation. Although initial models suggested that mood was particularly positive when self-appraisal and reflected appraisal were simultaneously elevated, this pattern no longer remained evident after accounting for nonlinear effects of self-appraisal. Instead, the analyses indicated that the association between self-appraisal and mood was itself nonlinear. Together, these findings suggest that what initially appeared to reflect evaluative congruence may instead be attributable to the underlying structure of self-appraisal and reflected appraisal themselves rather than congruence or discrepancy per se.

This interpretation is broadly consistent with both self-esteem research and cognitive models of emotional vulnerability, which emphasize the importance of self-evaluations and perceived social evaluations for psychological wellbeing (Beck, 1991; Ingram, 1990). Individuals who evaluate themselves more negatively and who believe they are evaluated less positively by others tend to report lower wellbeing and greater psychological distress. From this perspective, moments characterized by less positive self-evaluations and perceived social evaluations may be more closely associated with lower mood than moments characterized merely by inconsistency between the two appraisal domains.

More broadly, these findings suggest that the emotional significance of self–other evaluative processes may lie less in achieving consistency between appraisals and more in the positivity or negativity of the appraisals themselves. From this perspective, individuals may tolerate a certain degree of mismatch between self-appraisals and reflected appraisals without substantial emotional costs, whereas negative self-evaluations and negative perceived social evaluations appear more closely linked to momentary mood. This interpretation shifts attention from evaluative congruence per se toward the content of self-relevant evaluations as a potential driver of emotional experience in everyday life.

Taken together, the present findings challenge a straightforward discrepancy-based interpretation of self–other evaluative dynamics. Although evaluative inconsistency was initially associated with lower mood, this association was not robust after accounting for the component appraisals directly. Rather than supporting discrepancy itself as an independent affective mechanism, the findings suggest that momentary mood may be associated more strongly with the valence and structure of self-appraisals and reflected appraisals than with discrepancies between them. Future research should further examine under which conditions evaluative discrepancies become psychologically meaningful and whether discrepancy plays a more prominent role in specific interpersonal contexts, longer-term adjustment processes, or clinical populations.

### 4.4 Limitations and future directions

Several limitations should be considered when interpreting the present findings. First, although the ecological momentary assessment design allowed examination of within-person associations, the temporal resolution may not fully capture the timescale at which self-appraisal, reflected appraisal, and mood influence one another. Because prompt intervals varied, lagged effects represent variable temporal distances, which may attenuate autoregressive and cross-lagged associations. The absence of robust lagged effects, particularly for discrepancy predicting later mood, may therefore reflect rapid regulatory processes unfolding within shorter intervals than those assessed here, rather than an absence of temporal persistence.

In addition, social context was operationalized as a binary distinction between being alone and being with others. While this distinction captures a fundamental shift in evaluative environment, it necessarily obscures heterogeneity across social situations, including differences in interaction modality (e.g., in-person vs. digital interaction), interaction partners, relational closeness, and evaluative relevance, all of which may differentially shape self–other evaluative dynamics. Prior research suggests that reflected appraisals are particularly influential when the evaluating other is personally meaningful to the self (Carlson et al., 2011; Carlson, 2016), a distinction that could not be captured by the present operationalization. Future research would therefore benefit from a more fine-grained characterization of social context that differentiates between types of social relationships and their evaluative significance.

Furthermore, discrepancy was operationalized as an absolute difference score, which obscures directionality and may conflate level and difference effects. Future research should distinguish between positive and negative discrepancies to clarify whether specific forms of misalignment carry differential affective consequences. In addition, the reflected appraisal items did not specify a particular social referent. Participants may therefore have based reflected appraisals either on generalized beliefs about how others see them or on evaluations inferred from currently present interaction partners. Consequently, the psychological meaning of reflected appraisal may have varied across alone and social moments.

The generalizability of the findings is limited by the composition of the sample, which primarily consisted of young university students and required ownership of an Android smartphone for study participation. The observed dynamics may therefore not generalize to older populations, individuals with different educational backgrounds, or users of other mobile platforms. Future research should examine whether similar momentary self–other evaluative processes emerge in more diverse and clinically relevant populations. Finally, although within-person and lagged modeling approaches were employed, the observational nature of the data precludes strong causal inference. Experimental manipulations of social context or social feedback will be necessary to establish the mechanisms underlying the observed associations.

## 5 Conclusion

By examining self-appraisal and reflected appraisal simultaneously in daily life, the present study offers insight into how internal and socially inferred self-evaluations are linked in everyday experience. Self- and reflected appraisals were closely aligned within moments highlighting the strong correspondence between how individuals evaluate themselves and how they believe they are evaluated by others. Social context selectively shaped this association, suggesting that the presence of others may increase the salience of socially evaluative self-processing. However, the findings also indicate that self–other evaluative coupling does not simply increase whenever individuals are more socially engaged than usual, suggesting that the role of social context is more nuanced than a purely situational account would imply. At the same time, the findings suggest that the affective significance of self–other evaluative processes may reside less in the correspondence between evaluations than in how positively or negatively individuals evaluate themselves and believe they are evaluated by others. Individuals may therefore be able to tolerate moderate discrepancies between self-appraisals and reflected appraisals without substantial immediate emotional consequences.

Taken together, the findings suggest that everyday self-evaluation is shaped by an ongoing interplay between internal self-views and socially inferred evaluations. By integrating momentary, contextual, temporal, and affective processes, the present study contributes to a more nuanced understanding of how the social self is constructed and maintained in daily life.

## Data Availability

The raw data supporting the conclusions of this article will be made available by the authors, without undue reservation.
