# Supplementary material for: Identity in interaction: momentary dynamics of self-appraisal and reflected appraisal
Source: Front Psychol. 2026 Jul 15;17:1814576. doi: 10.3389/fpsyg.2026.1814576 (PMC13415972; doi:10.3389/fpsyg.2026.1814576)
Supplement: Supplementary file 1 [file Data_Sheet_1.PDF]

Supplementary Material

1 Supplementary Material[1]

1.1 Used Scales and their English translation

Situativer Gedanke (Bryant et al., 2013)
Ich habe gerade über mich nachgedacht
Ja/nein
Ich habe gerade über eine/andere Person(en) nachgedacht
Ja/nein
Spontanous thought (Bryant et al., 2013)
"I was just thinking about myself."
yes/no
"I was just thinking about (an)other person(s)."
yes/no

Stimmung (Wilhelm & Schoebi, 2007)
Im Moment fühle ich mich …
sehr unruhig [0 - 1 - 2 - 3 - 4 - 5 - 6] sehr ruhig
sehr angespannt [0 - 1 - 2 - 3 - 4 - 5 - 6] sehr entspannt
sehr müde [0 - 1 - 2 - 3 - 4 - 5 - 6] sehr wach
sehr energielos [0 - 1 - 2 - 3 - 4 - 5 - 6] sehr energiegeladen
Mood (Wilhelm & Schoebi, 2007)
At this moment I feel …
very agitated [0 - 1 - 2 - 3 - 4 - 5 - 6] very calm
very tensed [0 - 1 - 2 - 3 - 4 - 5 - 6] very relaxed
very tired [0 - 1 - 2 - 3 - 4 - 5 - 6] very awake
without energy [0 - 1 - 2 - 3 - 4 - 5 - 6] full of energy

Positiver and negativer Affekt (Simons et al., 2020)
Im Moment fühle ich mich …
fröhlich [0 - 1 - 2 - 3 - 4 - 5 - 6]
zufrieden [0 - 1 - 2 - 3 - 4 - 5 - 6]
glücklich [0 - 1 - 2 - 3 - 4 - 5 - 6]
unsicher [0 - 1 - 2 - 3 - 4 - 5 - 6]
ängstlich [0 - 1 - 2 - 3 - 4 - 5 - 6]
niedergeschlagen [0 - 1 - 2 - 3 - 4 - 5 - 6]
schuldig [0 - 1 - 2 - 3 - 4 - 5 - 6]
Positive and negative affect (Simons et al., 2020)
At this moment I feel …

[1] Scales not analyzed in the present article are included for documentation and transparency.

cheerful [0 - 1 - 2 - 3 - 4 - 5 - 6]
content [0 - 1 - 2 - 3 - 4 - 5 - 6]
happy [0 - 1 - 2 - 3 - 4 - 5 - 6]
insecure [0 - 1 - 2 - 3 - 4 - 5 - 6]
afraid [0 - 1 - 2 - 3 - 4 - 5 - 6]
down [0 - 1 - 2 - 3 - 4 - 5 - 6]
guilty [0 - 1 - 2 - 3 - 4 - 5 - 6]

Selbsteinschätzung (Collani & Herzberg, 2003; Santangelo, Koenig, et al., 2017)
Wie sehen Sie sich selbst in diesem Moment?
Trifft gar nicht zu – Trifft vollkommen zu [0 - 1 - 2 - 3 - 4 - 5 - 6 - 7 - 8 - 9]

- Ich bin mit mir selbst zufrieden.
- Ich denke, dass ich gar nichts tauge.
- Ich halte mich für einen Versager.
- Ich halte mich für einen wertvollen Menschen.

Self-appraisal (Collani & Herzberg, 2003; Santangelo, Koenig, et al., 2017)
At the moment
strongly agree – strongly disagree [0 - 1 - 2 - 3 - 4 - 5 - 6 - 7 - 8 - 9]

- I am satisfied with myself.
- I think I am no good at all.
- I am inclined to feel that I am a failure.
- I feel that I am a person of worth.

Perspektivwechsel (Collani & Herzberg, 2003; Santangelo, Koenig, et al., 2017)
Wie würden andere Menschen Sie in diesem Moment bewerten?
Trifft gar nicht zu – Trifft vollkommen zu [0 - 1 - 2 - 3 - 4 - 5 - 6 - 7 - 8 - 9]

- Andere Menschen sind zufrieden mit mir.
- Andere Menschen denken, dass ich gar nichts tauge.
- Andere Menschen halten mich für einen Versager.
- Andere halten mich für einen wertvollen Menschen.

Reflected Appraisal (Collani & Herzberg, 2003; Santangelo, Koenig, et al., 2017)
How do others see you at this moment?
At this moment
strongly agree – strongly disagree [0 - 1 - 2 - 3 - 4 - 5 - 6 - 7 - 8 - 9]

- Others are satisfied with me.~~I am satisfied with myself.~~
- Others ~~I~~ think I am no good at all.
- Others are~~I am~~ inclined to feel that I am a failure.
- Others consider me ~~I feel~~ ~~that I am~~ a person of worth.

Situativer sozialer Kontext
Ich bin grade…
… alleine.
… mit anderen Menschen in Kontakt.
Social Context
At this moment I am …
… alone.
… in contact with others.

2 Comparison of fixed-effect estimates from primary random-intercept models and corresponding random-slope specifications

The primary analyses reported in the manuscript were estimated using random-intercept models. During model development, models including random slopes for the focal within-person predictors in the central self-appraisal (SA) and reflected appraisal (RA) analyses were also specified. Specifically, random slopes were estimated for the within-person association between SA and RA across participants.

The random-slope models converged successfully using the bobyqa optimizer. However, singular-fit warnings were observed, reflecting near-zero variance estimates for some random-effects parameters. Consequently, the more parsimonious random-intercept models were retained as the primary analyses. For transparency, the corresponding random-slope models are presented below. Importantly, fixed-effect estimates remained substantively unchanged across model specifications, indicating that the reported findings were not dependent on the choice of random-effects structure.

Table 2
Comparison of fixed-effect estimates from primary random-intercept models and corresponding random-slope specifications

Outcome	Predictor	Random intercept (b)	Random slope (b)
RA	SA	0.52	0.51
	RA(t-1)	0.14	0.11
SA	RA	0.60	0.59
	SA(t−1)	0.17	0.15

Note. Primary analyses were estimated using random-intercept models. The random-slope models shown here correspond to alternative model specifications considered during model development but not retained because of singular-fit warnings. Fixed-effect estimates remained substantively unchanged across specifications.
