# Supplementary material for: Identity in interaction: momentary dynamics of self-appraisal and reflected appraisal
Source: Front Psychol. 2026 Jul 15;17:1814576. doi: 10.3389/fpsyg.2026.1814576 (PMC13415972; doi:10.3389/fpsyg.2026.1814576)
Supplement: Supplementary file 2 [file Supplementary_file_1.docx]

Supplementary Material

# Comparison of fixed-effect estimates from primary random-intercept models and corresponding random-slope specifications

The primary analyses reported in the manuscript were estimated using random-intercept models. During model development, models including random slopes for the focal within-person predictors in the central self-appraisal (SA) and reflected appraisal (RA) analyses were also specified. Specifically, random slopes were estimated for the within-person association between SA and RA across participants.

The random-slope models converged successfully using the bobyqa optimizer. However, singular-fit warnings were observed, reflecting near-zero variance estimates for some random-effects parameters. Consequently, the more parsimonious random-intercept models were retained as the primary analyses. For transparency, the corresponding random-slope models are presented below. Importantly, fixed-effect estimates remained substantively unchanged across model specifications, indicating that the reported findings were not dependent on the choice of random-effects structure.

**Table 2
Comparison of fixed-effect estimates from primary random-intercept models and corresponding random-slope specifications**

| **Outcome** | **Predictor** | **Random intercept (b)** | **Random slope (b)** |
| --- | --- | --- | --- |
| RA | SA | 0.52 | 0.51 |
|  | RA(t-1) | 0.14 | 0.11 |
| SA | RA | 0.60 | 0.59 |
|  | SA(t−1) | 0.17 | 0.15 |

*Note.* Primary analyses were estimated using random-intercept models. The random-slope models shown here correspond to alternative model specifications considered during model development but not retained because of singular-fit warnings. Fixed-effect estimates remained substantively unchanged across specifications.
